# Characterizing Subsurface Environments Using Borehole Magnetic Gradiometry

**DOI:** 10.3390/s25010171

**Published:** 2024-12-31

**Authors:** Mohammad Forman Asgharzadeh, Hasan Ghasemzadeh, Ralph von Frese, Kamran Ighani

**Affiliations:** 1 School of Earth Sciences, Ohio State University, Columbus, OH 43210, USA; 2 Department of Civil Engineering, K. N. Toosi University of Technology, Tehran 19967-15433, Iran; ghasemzadeh@kntu.ac.ir (H.G.); k.ighani@email.kntu.ac.ir (K.I.)

**Keywords:** magnetism, gradiometry, numerical modeling, borehole instrumentation

## Abstract

Forward modeling the magnetic effects of an inferred source is the basis of magnetic anomaly inversion for estimating subsurface magnetization parameters. This study uses numerical least-squares Gauss–Legendre quadrature (GLQ) integration to evaluate the magnetic potential, anomaly, and gradient components of a cylindrical prism element. Relative to previous studies, it quantifies for the first time the magnetic gradient components, enabling their applications in the interpretation of cylindrical bodies. A comparison of this method to other methods of evaluating the vertical component of the magnetic field associated with a full cylinder shows that it has comparable to improved performance in computational accuracy and speed. Based on the developed theory, a conceptual design is presented for an instrument to measure the magnetic gradient effects of subsurface material in the vicinity of a borehole. The significance of this instrument relative to conventional borehole magnetometers is in its ability to determine the azimuthal directions of magnetic sources within the borehole environment.

## 1. Introduction

**Figure A1:**
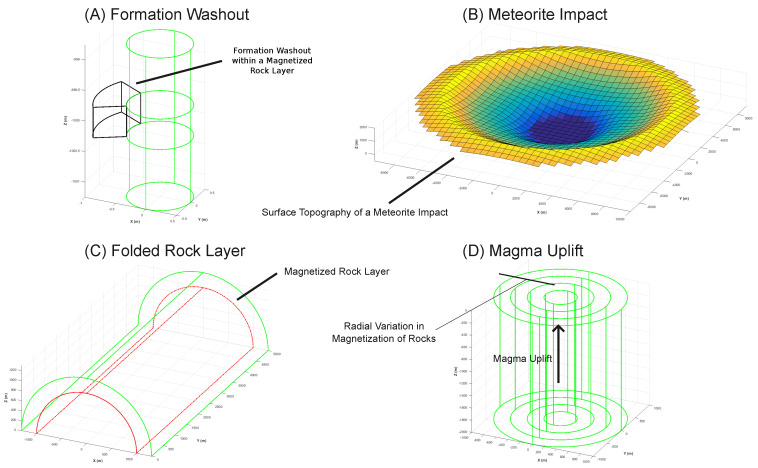
Application of cylindrical magnetic modeling in (**A**) formation washouts in magnetized rock layers, (**B**) meteorite impacts, (**C**) folded magnetized rock layers, and (**D**) cylindrical conduits transferring magmatic material upward from deep Earth chambers.

Magnetic data collected from within boreholes and at surface, airborne, and satellite altitudes contain valuable information regarding the distribution of magnetic bodies within the subsurface. The inference of source body geometry from collected data requires numerical modeling in natural coordinate systems. The modeling of the magnetic effects in a cylindrical coordinate system finds applications in areas such as estimating the magnetic effects of borehole washouts within magnetized formations, meteorite impacts, folded magnetized rock layers, cylindrical crustal conduits transferring magmatic material, and other cylindrical shaped geological features (Figure A1 in Appendix A).

Several studies have considered the modeling of the effects of homogenously magnetized bodies in cylindrical coordinates. For example, Ref. [1] modeled the vertical magnetic field component of the infinite horizontal, semi-infinite vertical, and finite vertical magnetic cylinder under uniform vertical magnetization. Ref. [2] quantified the use of Legendre polynomials for computing the magnetic potential and vector field of a vertical cylinder with spatially uniform but arbitrarily directed magnetization. To extend the Refs. [3,4] studies, Ref. [5] used elliptical integrals in a closed form to model the magnetic field effects of the finite cylinder with uniform but arbitrarily directed magnetization. On the other hand, Ref. [6] used the vector magnetic potential to compute the magnetic vector field of a cylindrical tile or prism with uniform magnetization. Additionally, Ref. [7] invoked the scalar magnetic potential to compute the magnetic vector field of a homogeneously magnetized cylindrical prism.

The above studies generally consider the scalar potential and/or vector field components of a uniform magnetic prism or cylinder, often in the context of complex and laborious computations. However, none of these studies provide an account for the cylinder’s magnetic-tensor gradient field components. Accordingly, the present study quantifies these more extended attributes of the magnetic effects for the uniformly magnetized cylindrical prism. Relative to vector field data, gradient data commonly provide enhanced detail concerning the magnetic anomaly’s spatial variations.

In the sections below, we first quantitatively model the magnetic effects of a uniformly magnetized cylindrical prism. We then present a workflow to implement the numerical GLQ integration in a cylindrical coordinate system. Next, we extend our formulation to model the magnetic effects of a cylindrical prism as well as a formation washout under arbitrary magnetization conditions. We then present an instrument design concept for measuring the magnetic gradient field in a borehole environment. Next, we compare our developed equations to other computational methods and discuss the applicational significance of the proposed instrument design to conventional borehole magnetometry. Finally, we make a number of suggestions for further use of the GLQ modeling method developed in this research.

## 2. Materials and Methods

### 2.1. Magnetic Effects for the Cylindrical Prism

**Figure A2:**
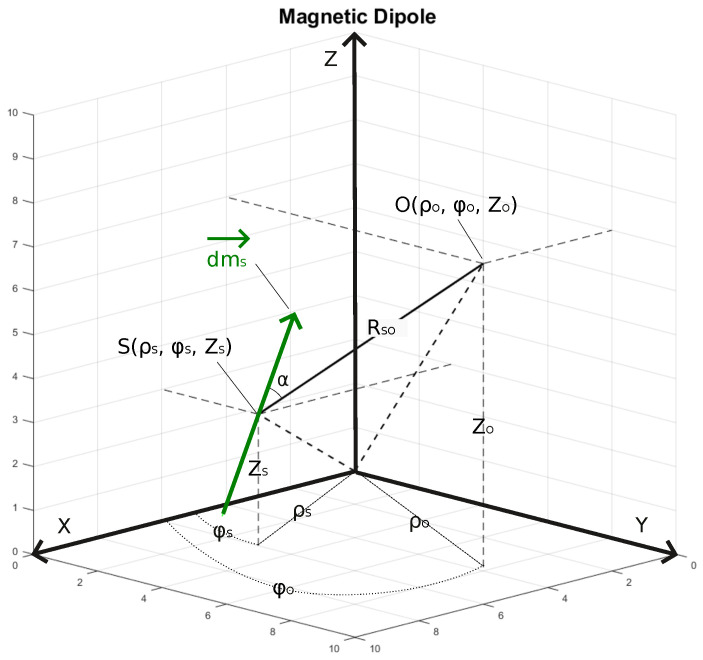
The magnetic moment vector ${\mathrm{dm}}_{S}$ located at source point $S(\rho_{S},\phi_{S},Z_{S})$ produces the magnetic potential at observation point $O(\rho_{O},\phi_{O},Z_{O})$.

Consider the local co-registered (XYZ)-Cartesian and (ρϕZ)-cylindrical coordinate systems (Figure A2) with the magnetic moment vector ${\mathrm{dm}}_{S}\left({dm_{S}}_{X},{dm_{S}}_{Y},{dm_{S}}_{Z}\right)$ located at the source point $S(\rho_{S},\phi_{S},Z_{S})$. The length of the vector $R_{\mathrm{SO}}\left({R_{SO}}_{X},{R_{SO}}_{Y},{R_{SO}}_{Z}\right)$ connecting *S* to an observation point $O(\rho_{O},\phi_{O},Z_{O})$ in the local cylindrical coordinate system is

(1) $$R_{SO}={\left[{\rho_{O}}^{2}+{\rho_{S}}^{2}-2\rho_{O}\rho_{S}cos\left(\phi_{O}-\phi_{S}\right)+{\left(Z_{O}-Z_{S}\right)}^{2}\right]}^{\frac{1}{2}}.$$

The magnetic moment’s scalar magnetic potential $d\psi_{SO}$ may be approximated at *O* [8] by

(2) $$d\psi_{SO}=-\frac{\mu_{0}}{4\pi }\frac{{dm_{S}}_{X}{R_{SO}}_{X}+{dm_{S}}_{Y}{R_{SO}}_{Y}+{dm_{S}}_{Z}{R_{SO}}_{Z}}{R_{SO}^{3}},$$

where ${R_{SO}}_{X}$, ${R_{SO}}_{Y}$, and ${R_{SO}}_{Z}$ are the Cartesian components of $R_{SO}$ that may be expressed in cylindrical coordinates by

(3) $$\left(\begin{array}{c} {R_{SO}}_{X}\\ {R_{SO}}_{Y}\\ {R_{SO}}_{Z} \end{array}\right)=\left(\begin{array}{c} {R_{O}}_{X}-{R_{S}}_{X}\\ {R_{O}}_{Y}-{R_{S}}_{Y}\\ {R_{O}}_{Z}-{R_{S}}_{Z} \end{array}\right)=\left(\begin{array}{c} \rho_{O}cos\phi_{O}-\rho_{S}cos\phi_{S}\\ \rho_{O}sin\phi_{O}-\rho_{S}sin\phi_{S}\\ Z_{O}-Z_{S} \end{array}\right),$$

**Figure 1:**
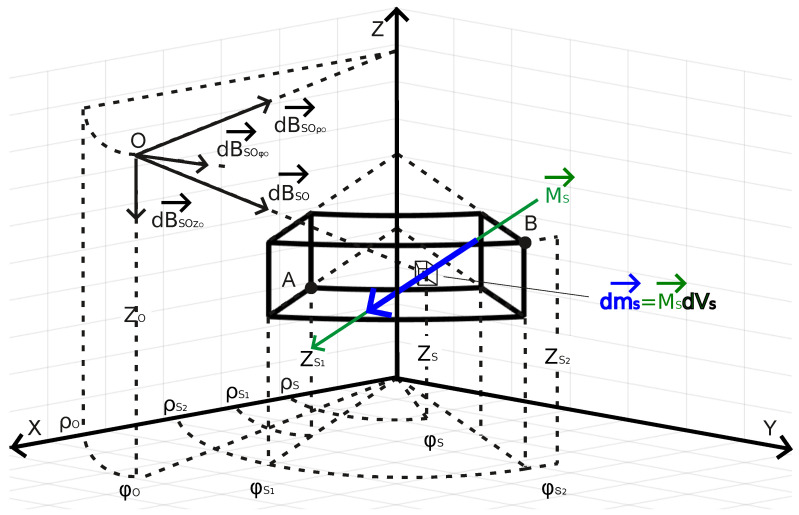
The cylindrical ρϕZ coordinate system for modeling at the observation point $O(\rho_{O},\phi_{O},Z_{O})$ the magnetic effects of a cylindrical prism by integrating the differential source magnetic moment ${\mathrm{dm}}_{S}$ through the volume of the prism with bottom and top surfaces that include the corner points $A({\rho_{S}}_{1},{\phi_{S}}_{1},{Z_{S}}_{1})$ and $B({\rho_{S}}_{2},{\phi_{S}}_{2},{Z_{S}}_{2})$. The Cartesian perspective is given by the superposed, co-registered Cartesian XYZ coordinate system axes.

In practice, the differential potential in Equation (2) must be expressed in terms of magnetization $M_{S}\left({M_{S}}_{X},{M_{S}}_{Y},{M_{S}}_{Z}\right)=\frac{{\mathrm{dm}}_{S}\left({dm_{S}}_{X},{dm_{S}}_{Y},{dm_{S}}_{Z}\right)}{dV_{S}}$. Here, the differential volume in cylindrical coordinates (Figure 1) is $dV_{S}=\rho_{S}d\rho_{S}d\phi_{S}dZ_{S}$, so Equation (2) becomes

(4) $$\begin{array}{cc} \; & d\psi_{SO}=-\frac{\mu_{0}}{4\pi }\\ \; & \frac{{M_{S}}_{X}\left(\rho_{O}cos\phi_{O}-\rho_{S}cos\phi_{S}\right)+{M_{S}}_{Y}\left(\rho_{O}sin\phi_{O}-\rho_{S}sin\phi_{S}\right)+{M_{S}}_{Z}\left(Z_{O}-Z_{S}\right)}{R_{SO}^{3}}\\ \; & \rho_{S}d\rho_{S}d\phi_{S}dZ_{S}. \end{array}$$

Equation (4) is the basis for computing the magnetic effects of the cylindrical prism at observation point *O*. To model the magnetic effects of differential volumes in other coordinate systems (Cartesian, spherical, elliptical, etc.), care must be taken to express $dV_{S}$ in the appropriate coordinate system.

The magnetic vector field ${\mathrm{dB}}_{\mathrm{SO}}\left({dB_{SO}}_{\rho_{O}},{dB_{SO}}_{\phi_{O}},{dB_{SO}}_{Z_{O}}\right)$ for $d\psi_{SO}$ is given by its spatial gradient.

(5) $${\mathrm{dB}}_{\mathrm{SO}}=\nabla d\psi_{SO}=\frac{\partial (d\psi_{SO})}{\partial \rho_{O}}{\hat{\rho }}_{O}+\frac{1}{\rho_{O}}\frac{\partial (d\psi_{SO})}{\partial \phi_{O}}{\hat{\phi }}_{O}+\frac{\partial (d\psi_{SO})}{\partial Z_{O}}{\hat{Z}}_{O}.$$

Equation (5) in component form is

(6) $$\left(\begin{array}{c} {dB_{SO}}_{\rho_{O}}\\ {dB_{SO}}_{\phi_{O}}\\ {dB_{SO}}_{Z_{O}} \end{array}\right)=-\frac{\mu_{0}\rho_{S}}{4\pi }\cdot \left(\begin{array}{c} N_{1}\\ N_{2}\\ N_{3} \end{array}\right)\cdot \frac{1}{D}d\rho_{S}d\phi_{S}dZ_{S},$$

where $N_{1}$, $N_{2}$, $N_{3}$ and *D* are defined in Table A1 (in Appendix B). Furthermore, the spatial gradients of the magnetic field components in Equations (6) involve the elements of the 3×3 matrix given by

(7) $$\nabla dB_{SO}=\left(\begin{array}{ccc} {{dB_{SO}}_{\rho_{O}}}_{\rho_{O}} & {{dB_{SO}}_{\rho_{O}}}_{\phi_{O}} & {{dB_{SO}}_{\rho_{O}}}_{Z_{O}}\\ {{dB_{SO}}_{\phi_{O}}}_{\rho_{O}} & {{dB_{SO}}_{\phi_{O}}}_{\phi_{O}} & {{dB_{SO}}_{\phi_{O}}}_{Z_{O}}\\ {{dB_{SO}}_{Z_{O}}}_{\rho_{O}} & {{dB_{SO}}_{Z_{O}}}_{\phi_{O}} & {{dB_{SO}}_{Z_{O}}}_{Z_{O}} \end{array}\right),$$

where ${{dB_{SO}}_{\rho_{O}}}_{\rho_{O}}$ is the spatial gradient of ${dB_{SO}}_{\rho_{O}}$ in the $\rho_{o}$-radial direction, ${{dB_{SO}}_{\rho_{O}}}_{\phi_{O}}$ is the spatial gradient of ${dB_{SO}}_{\rho_{O}}$ in the $\phi_{o}$-longitudinal direction, and so on. These elements are formulated in Table A2 with the related partial differentials and supplemental details listed in Table A3 and Table A4, respectively.

The magnetic potential ($\psi_{O}$), vector ($B_{O_{m}}$), and gradient tensor ($B_{{O_{m}}_{n}}$) components at point *O*, due to the uniform magnetization $M_{S}$, from ${\rho_{S}}_{1}$ to ${\rho_{S}}_{2}$, ${\phi_{S}}_{1}$ to ${\phi_{S}}_{2}$ and ${Z_{S}}_{1}$ to ${Z_{S}}_{2}$ are, respectively, given by

(8a) $$\psi_{O}=\int_{V}d\psi_{SO}=\int_{Z_{S}={Z_{S}}_{1}}^{{Z_{S}}_{2}}\int_{\phi_{S}={\phi_{S}}_{1}}^{{\phi_{S}}_{2}}\int_{\rho_{S}={\rho_{S}}_{1}}^{{\rho_{S}}_{2}}d\psi_{SO},$$

(8b) $${B_{O}}_{m}=\int_{V}{dB_{SO}}_{m}=\int_{Z_{S}={Z_{S}}_{1}}^{{Z_{S}}_{2}}\int_{\phi_{S}={\phi_{S}}_{1}}^{{\phi_{S}}_{2}}\int_{\rho_{S}={\rho_{S}}_{1}}^{{\rho_{S}}_{2}}{dB_{SO}}_{m},$$

(8c) $${{B_{O}}_{m}}_{n}=\int_{V}{{dB_{SO}}_{m}}_{n}=\int_{Z_{S}={Z_{S}}_{1}}^{{Z_{S}}_{2}}\int_{\phi_{S}={\phi_{S}}_{1}}^{{\phi_{S}}_{2}}\int_{\rho_{S}={\rho_{S}}_{1}}^{{\rho_{S}}_{2}}{{dB_{SO}}_{m}}_{n},$$

where *m* is the $\rho_{O}$, $\phi_{O}$, or $Z_{O}$ component of the vector- and tensor-field intensities and *n* is the $\rho_{O}$, $\phi_{O}$, or $Z_{O}$ direction along which the gradient is taken.

The triple integrals in Equations (8a)–(8c) clearly involve computationally complex and laborious analytical solutions. However, their solutions may be elegantly generalized as numerical least-squares approximations by Gauss–Legendre quadrature integration (e.g., [9,10,11,12]), given by

(9) $$\begin{array}{cc} \; & \int_{Z_{S}={Z_{S}}_{1}}^{{Z_{S}}_{2}}\int_{\phi_{S}={\phi_{S}}_{1}}^{{\phi_{S}}_{2}}\int_{\rho_{S}={\rho_{S}}_{1}}^{{\rho_{S}}_{2}}\left[f\left(\rho_{O},\phi_{O},Z_{O},\rho_{S},\phi_{S},Z_{S}\right)\right]d\rho_{S}d\phi_{S}dZ_{S}≅\\ \; & \frac{\left({\rho_{S}}_{2}-{\rho_{S}}_{1}\right)\left({\phi_{S}}_{2}-{\phi_{S}}_{1}\right)\left({Z_{S}}_{2}-{Z_{S}}_{1}\right)}{8}\\ \; & \sum_{n_{k}=1}^{K}\sum_{n_{j}=1}^{J}\sum_{n_{i}=1}^{I}\left[f\left(\rho_{O},\phi_{O},Z_{O},{\hat{\rho }}_{n_{i}},{\hat{\phi }}_{n_{j}},{\hat{Z}}_{n_{k}}\right)\right]A_{n_{i}}A_{n_{j}}A_{n_{k}}. \end{array}$$

Here, the square bracketed *f*-functions are generalizations of the appropriate integrands (e.g., Equations (4), (6) and (7)) in observation and source point coordinates, and the Gauss–Legendre coefficients $A_{n_{i}}$, $A_{n_{j}}$, and $A_{n_{k}}$ correspond to the $\rho_{n_{i}}$, $\phi_{n_{j}}$, and $Z_{n_{k}}$ coordinates of the *n*-th Gaussian node within the interval (−1,1). To transform the integration from the unit interval into the ${\hat{\rho }}_{n_{i}}$, ${\hat{\phi }}_{n_{j}}$, and ${\hat{Z}}_{n_{k}}$ coordinates within the source body requires scaling the body point coordinates as

(10a) $${\hat{\rho }}_{n_{i}}=\frac{\rho_{n_{i}}\left({\rho_{S}}_{2}-{\rho_{S}}_{1}\right)+\left({\rho_{S}}_{2}+{\rho_{S}}_{1}\right)}{2},$$

(10b) $${\hat{\phi }}_{n_{j}}=\frac{\phi_{n_{j}}\left({\phi_{S}}_{2}-{\phi_{S}}_{1}\right)+\left({\phi_{S}}_{2}+{\phi_{S}}_{1}\right)}{2},$$

and

(10c) $${\hat{Z}}_{n_{k}}=\frac{Z_{n_{k}}\left({Z_{S}}_{2}-{Z_{S}}_{1}\right)+\left({Z_{S}}_{2}+{Z_{S}}_{1}\right)}{2}.$$

Thus, Equation (9) computes the least squares magnetic effects of the cylindrical prism by summing at each observation point the GLQ-coefficient weighted magnetic effects of I×J×K equivalent point dipoles. The accuracies of the GLQ estimates in Equation (9) are directly controlled by the selected number of (I,J,K) nodes that, in turn, directly control the computation time. In practice, effective accuracy is achieved by selecting a node spacing that is smaller than the distance between the source nodes and the observation point (e.g., [10,11,13]). The accuracy of any selected number of nodes may be checked by comparing the result against the one from twice as many nodes to ensure that the difference is negligible.

To evaluate the magnetic effects of cylindrical bodies, the cylindrical coordinate system offers clear advantages. However, these effects are commonly observed by measurements registered in Cartesian coordinates (e.g., [14]). Here, the cylindrical-to-Cartesian coordinate transformations for the magnetic vector and tensor components at the observation point are given, respectively, by

(11) $$\left(\begin{array}{c} {B_{O}}_{X_{O}}\\ {B_{O}}_{Y_{O}}\\ {B_{O}}_{Z_{O}} \end{array}\right)=P\left(\phi_{O}\right)\left(\begin{array}{c} {B_{O}}_{\rho_{O}}\\ {B_{O}}_{\phi_{O}}\\ {B_{O}}_{Z_{O}} \end{array}\right),$$

and

(12) $$\begin{array}{ll} \left(\begin{array}{ccc} {{B_{O}}_{X_{O}}}_{X_{O}} & {{B_{O}}_{X_{O}}}_{Y_{O}} & {{B_{O}}_{X_{O}}}_{Z_{O}}\\ {{B_{O}}_{Y_{O}}}_{X_{O}} & {{B_{O}}_{Y_{O}}}_{Y_{O}} & {{B_{O}}_{Y_{O}}}_{Z_{O}}\\ {{B_{O}}_{Z_{O}}}_{X_{O}} & {{B_{O}}_{Z_{O}}}_{Y_{O}} & {{B_{O}}_{Z_{O}}}_{Z_{O}} \end{array}\right) & =\\ & P\left(\phi_{O}\right)\left(\begin{array}{ccc} {{B_{O}}_{\rho_{O}}}_{\rho_{O}} & {{B_{O}}_{\rho_{O}}}_{\phi_{O}} & {{B_{O}}_{\rho_{O}}}_{Z_{O}}\\ {{B_{O}}_{\phi_{O}}}_{\rho_{O}} & {{B_{O}}_{\phi_{O}}}_{\phi_{O}} & {{B_{O}}_{\phi_{O}}}_{Z_{O}}\\ {{B_{O}}_{Z_{O}}}_{\rho_{O}} & {{B_{O}}_{Z_{O}}}_{\phi_{O}} & {{B_{O}}_{Z_{O}}}_{Z_{O}} \end{array}\right)P^{T}\left(\phi_{O}\right), \end{array}$$

where $P^{T}\left(\phi_{O}\right)$ is the transpose of

(13) $$P\left(\phi_{O}\right)=\left(\begin{array}{ccc} cos(\phi_{O}) & -sin(\phi_{O}) & 0\\ sin(\phi_{O}) & cos(\phi_{O}) & 0\\ 0 & 0 & 1 \end{array}\right).$$

In the equations above, the source magnetization $M_{S}$ is the induced $M_{S,I}$ magnetization plus available remnant magnetization $M_{S,R}$ according to the vector sum

(14a) $$M_{S}=M_{S,I}+M_{S,R}=\chi_{S}H_{S}+M_{S,R},$$

with components that may be expressed in matrix format as

(14b) $$\left(\begin{array}{c} {M_{S}}_{X}\\ {M_{S}}_{Y}\\ {M_{S}}_{Z} \end{array}\right)=\left(\begin{array}{ccc} {\chi_{S}}_{XX} & {\chi_{S}}_{XY} & {\chi_{S}}_{XZ}\\ {\chi_{S}}_{YX} & {\chi_{S}}_{YY} & {\chi_{S}}_{YZ}\\ {\chi_{S}}_{ZX} & {\chi_{S}}_{ZY} & {\chi_{S}}_{ZZ} \end{array}\right)\cdot \left(\begin{array}{c} {H_{S}}_{X}\\ {H_{S}}_{Y}\\ {H_{S}}_{Z} \end{array}\right)+\left(\begin{array}{c} {M_{S,R}}_{X}\\ {M_{S,R}}_{Y}\\ {M_{S,R}}_{Z} \end{array}\right),$$

where $\chi_{S}$ is the source prism’s magnetic susceptibility tensor [15] by

(15) $$\chi_{S}=\left(\begin{array}{ccc} {\chi_{S}}_{XX} & {\chi_{S}}_{XY} & {\chi_{S}}_{XZ}\\ {\chi_{S}}_{YX} & {\chi_{S}}_{YY} & {\chi_{S}}_{YZ}\\ {\chi_{S}}_{ZX} & {\chi_{S}}_{ZY} & {\chi_{S}}_{ZZ} \end{array}\right),$$

and $H_{S}\left({H_{S}}_{X},{H_{S}}_{Y},{H_{S}}_{Z}\right)$ is the Earth’s magnetic field at the location of the prism. Equation (14b) models the induced and remnant magnetic effects individually or superposed at any observation point *O* on or outside the cylindrical prism’s surface.

### 2.2. Practical Implementation

Appendix C describes a workflow for computing the magnetic effects of a source at a series of prescribed observation points in any natural coordinate system. By adjusting to Equations (8a)–(8c), this workflow may be used to compute the magnetic effects of a cylindrically symmetric magnetic body. The workflow makes no reference to computational performance issues. Optimization may be achieved using parallel processing, Graphical Processing Unit (GPU) and other hardware and software technologies that are beyond the scope of this study.

## 3. Results

### 3.1. GLQ Magnetic Modeling of the Cylindrical Prism

**Figure 2:**
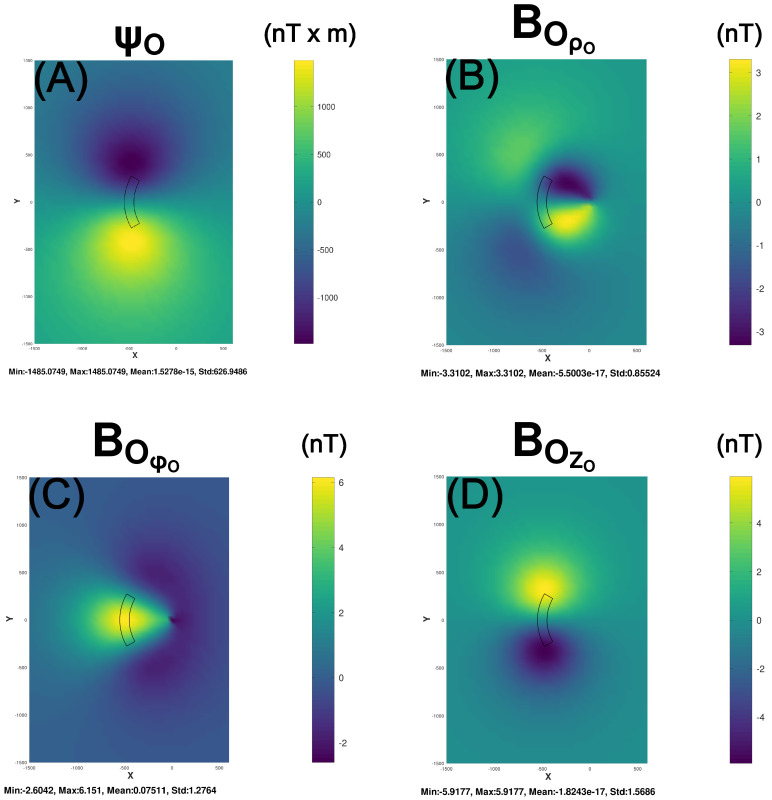
Maps (**A**–**D**) give the respective magnetic potential ($\psi_{O}$) and vector radial (${B_{O}}_{R_{O}}$), longitudinal (${B_{O}}_{\phi_{O}}$), and vertical (${B_{O}}_{Z_{O}}$) magnetic anomaly components of the cylindrical prism with ${\rho_{S}}_{1}=$ 450 m, ${\rho_{S}}_{2}=$ 550 m, ${\phi_{S}}_{1}=150{}^{\circ}$, ${\phi_{S}}_{2}=210{}^{\circ}$, ${Z_{S}}_{1}=-100$ m, and ${Z_{S}}_{2}=100$ m and magnetization $M_{S}(M_{S_{X}}=0.0,\;M_{S_{Y}}=1.0$ A/m, $M_{S_{Z}}=0.0)$ in cylindrical coordinates. The magnetic effects were modeled at a 500 m altitude for the cylindrical prism using I=2, J=8, and K=4 nodes. In the statistics of Figure 2, Figure 3, Figure 5, Figure A3, Figure A4, Figure A5 and Figure A6, $Me-N=M\times 10^{\left(-N\right)}$.

**Figure 3:**
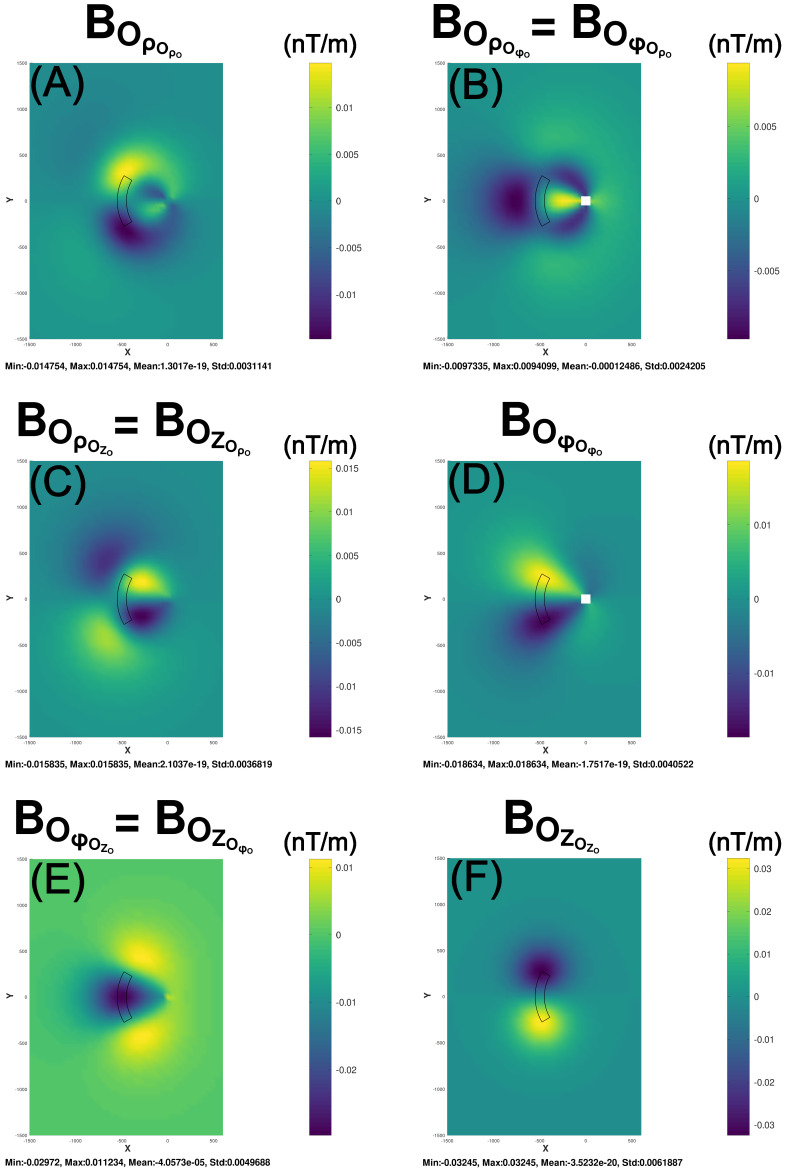
Maps (**A**–**F**) give the respective cylindrical coordinate magnetic gradient components for the cylindrical prism in Figure 2.

Figure 2 and Figure 3 illustrate the cylindrical coordinate magnetic effects at a 500 m altitude for the cylindrical volume element or prism with $M_{S}(M_{S_{X}}=0.0,\;M_{S_{Y}}=1.0$ A/m, $M_{S_{Z}}=0.0)$ and dimensions ${\rho_{S}}_{1}=450$ m to ${\rho_{S}}_{2}=550$ m, ${\phi_{S}}_{1}=150{}^{\circ}$ to ${\phi_{S}}_{2}=210{}^{\circ}$, and ${Z_{S}}_{1}=-100$ m to ${Z_{S}}_{2}=100$ m. These effects were GLQ-modeled using 2×8×4 nodes spanning the radial, azimuthal and axial dimensions, respectively, of the prism’s volume with the body point coordinates from Equation (10). Figure 2A–D, respectively, show the modeled magnetic $\psi_{O}$-potential and vector magnetic ${B_{O}}_{\rho_{O}}$-, ${B_{O}}_{\phi_{O}}$-, and ${B_{O}}_{Z_{O}}$- anomaly components.

**Figure A3:**
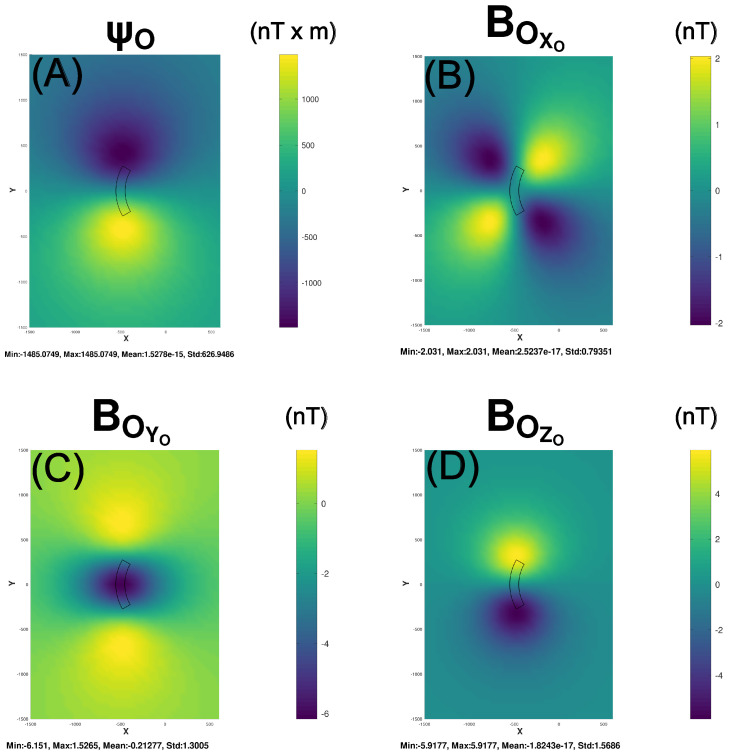
Maps (**A**–**D**) give the respective magnetic potential ($\psi_{O}$) and vector left–right (${B_{O}}_{X_{O}}$), top–bottom (${B_{O}}_{Y_{O}}$), and vertical (${B_{O}}_{Z_{O}}$) magnetic anomaly components for the cylindrical prism in Figure 2 in Cartesian coordinates. Note that the anomaly maps in Figures 2A and (**A**), as well as Figures 2D and (**D**), are the same for both cylindrical and Cartesian coordinate systems.

**Figure A4:**
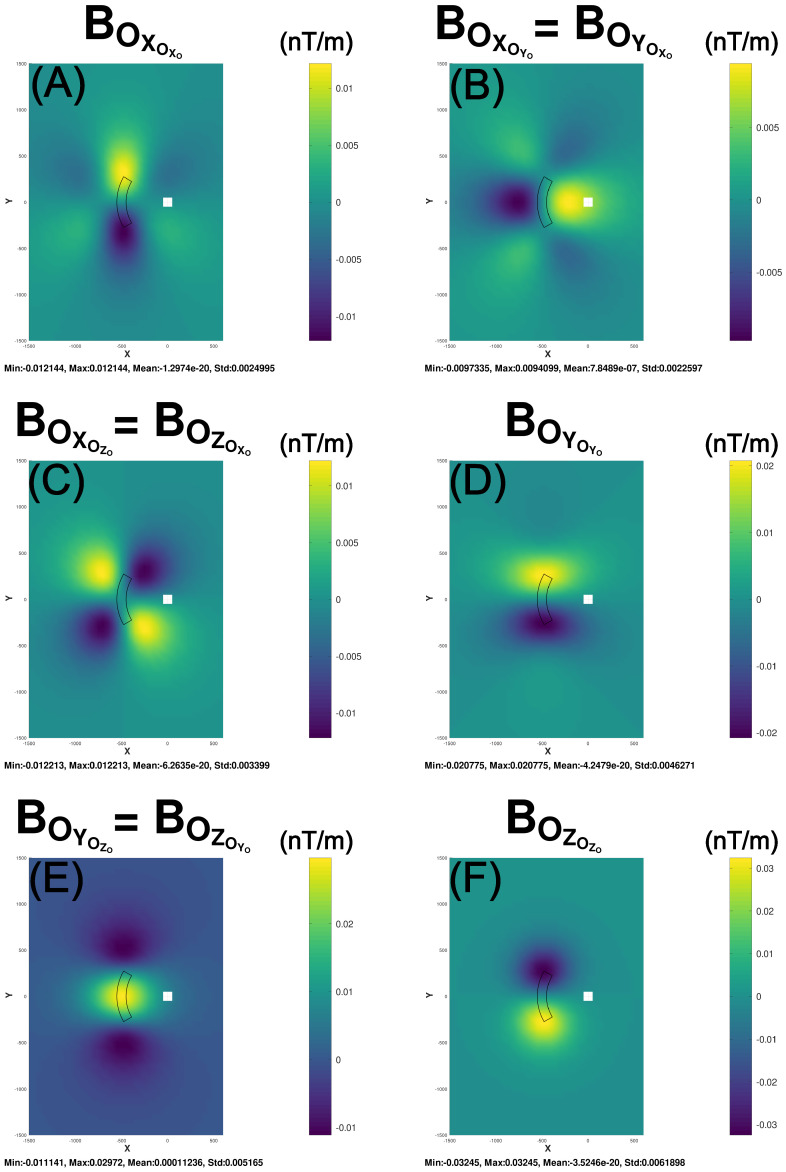
Maps (**A**–**F**) give the respective magnetic gradient components for the cylindrical prism in Figure 2 in Cartesian coordinates. Note that the anomaly maps in Figures 3F and (**F**) are the same for both cylindrical and Cartesian coordinate systems.

Figure 3 gives the magnetic gradient anomaly components from the three trace and three unique off-diagonal elements of the symmetric matrix in Equation (7). The magnetic effects of the cylindrical prism in Figure 2 and Figure 3 are, respectively, mapped in Cartesian coordinates in Figure A3 and Figure A4 via the transformations of Equations (11)–(13). Although cylindrical and Cartesian projections map equivalent spatial anomaly details, geophysical exploration applications, in practice, commonly utilize Cartesian mapping [14].

**Figure A5:**
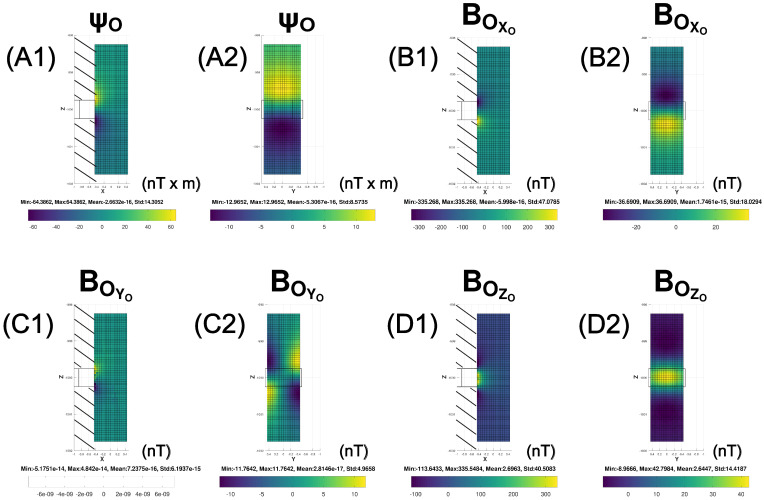
Magnetic potential ($\psi_{O}$), vector ${B_{O}}_{X_{O}}$, ${B_{O}}_{Y_{O}}$, and ${B_{O}}_{Z_{O}}$ magnetic anomaly components in the respective X, Y, and Z directions for a formation washout with radius $\rho_{Borehole}=0.5$ m. The washout extends from ${\rho_{S}}_{1}=0.5$ m to ${\rho_{S}}_{2}=1.0$ m, ${\phi_{S}}_{1}=150{}^{\circ}$ to ${\phi_{S}}_{2}=210{}^{\circ}$, and ${Z_{S}}_{1}=-1000.25$ m to ${Z_{S}}_{2}=-999.75$ m along the radial, azimuthal, and axial dimensions, respectively, of the borehole within a magnetized layer of magnetization $M_{S}(M_{S_{X}}=0.0,,M_{S_{Y}}=0.0,\;M_{S_{Z}}=1.0$ A/m). The magnetic effects were modeled along the wellbore’s central XZ (**A1**–**D1**) and YZ (**A2**–**D2**) planes using I=5, J=21, and K=20 nodes.

**Figure A6:**
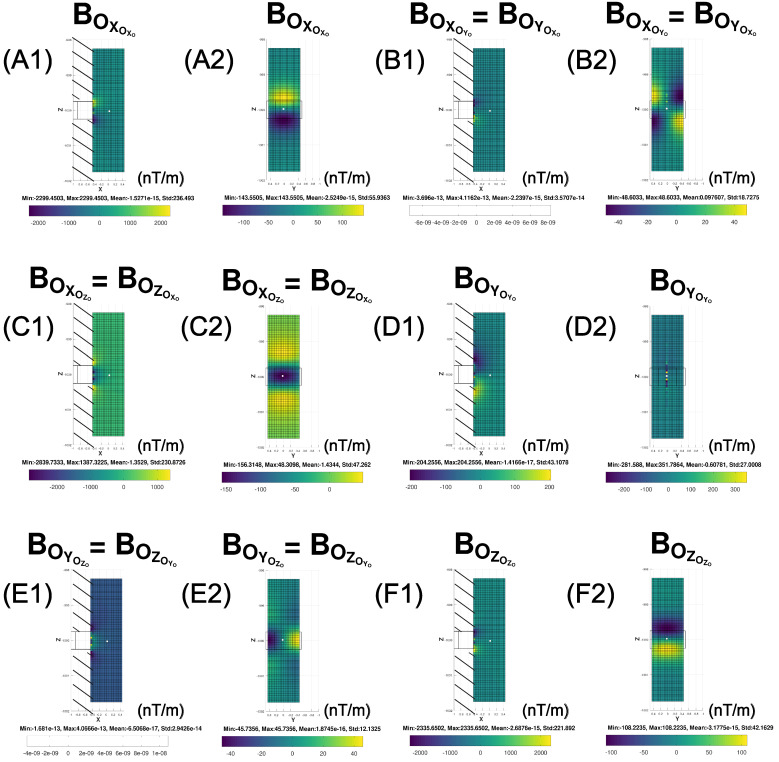
Magnetic gradient components for the formation washout in Figure A5 in Cartesian coordinates. The magnetic effects were modeled along the wellbore’s central XZ (**A1**–**F1**) and YZ (**A2**–**F2**) planes.

We next extend the application of gravity GLQ modeling [14] to model the magnetic effects of a hypothetical formation washout within a magnetized layer. Figure A5 and Figure A6 present a washout’s Cartesian coordinate magnetic effects along the respective central XZ and YZ planes of the borehole with a radius of $\rho_{Borehole}=0.5$ m. The washout extends from ${\rho_{S}}_{1}=0.5$ m to ${\rho_{S}}_{2}=1.0$ m, ${\phi_{S}}_{1}=150{}^{\circ}$ to ${\phi_{S}}_{2}=210{}^{\circ}$, and ${Z_{S}}_{1}=-1000.25$ m to ${Z_{S}}_{2}=-999.75$ m along the radial, azimuthal, and axial dimensions, respectively, of the borehole. The magnetization of the washout’s confining formation is $M_{S}(M_{S_{X}}=0.0,\;$$M_{S_{Y}}=0.0,\;M_{S_{Z}}=1.0$ A/m).

These effects were initially GLQ-modeled in cylindrical coordinates via Equations (8a)–(8c) using 5×21×20=2100 nodes, and subsequently projected into Cartesian coordinates via Equations (11)–(13). The figures indicate that ${B_{O}}_{Y_{O}}$, ${{B_{O}}_{X_{O}}}_{Y_{O}}={{B_{O}}_{Y_{O}}}_{X_{O}}$, and ${{B_{O}}_{Y_{O}}}_{Z_{O}}={{B_{O}}_{Z_{O}}}_{Y_{O}}$ show no variation (small variations on the order of $10^{-13}$ and less are the result of numerical errors) in contrast to the other components that vary strongly across the XZ-plane, whereas all components vary strongly across the YZ-plane.

### 3.2. Design of a Magnetic Probe for Borehole Magnetometry

**Figure 4:**
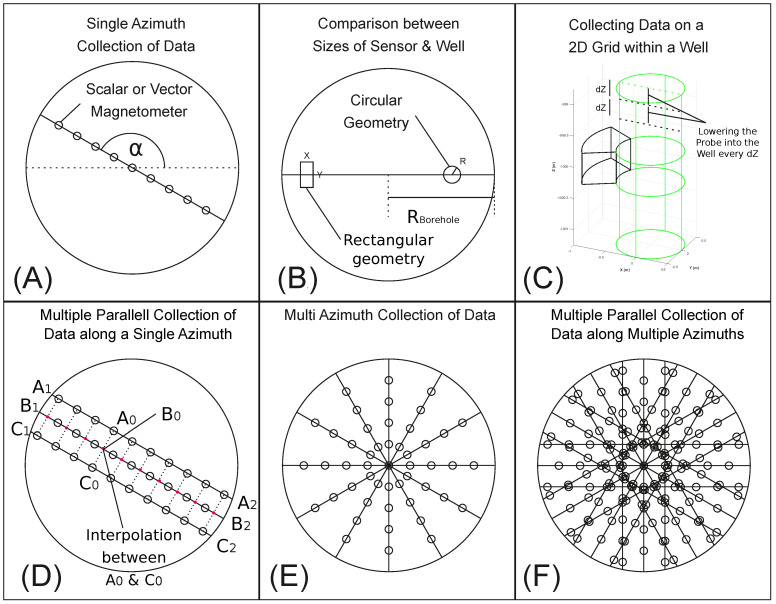
(**A**) Planar view of borehole magnetometer sensors across a single α azimuth. (**B**) The size and shape of a sensor determine how many measurements may be made at every $Z_{i}$ depth across the diameter of the borehole. (**C**) Three–dimensional view of azimuthal magnetic data collected within the borehole. The spatial density of data may be increased by collecting data (**D**) along multiple parallel rods (**E**) across multiple azimuths or (**F**) in some combination thereof.

In this section, we present a conceptual design of a borehole magnetometer probe that offers better characterization of magnetic sources within the vicinity of the borehole walls. Collecting magnetic data at several points along the diameter of a borehole will assist with understanding the horizontal variations of the magnetic field within the borehole (Figure 4A). Table A5 lists readily available magnetometers for collecting total intensity scalar or three-component vector magnetic data within the borehole environment. The horizontal XY dimensions of a magnetometer relative to the wellbore’s diameter dictate the number of measurements that can be made within the well (Figure 4B).

Among the fluxgate magnetometers in Table A5, the FEREX 4.035, FGM650 and VSM4 sensors with diameters less than 0.04 m are effective candidates for collecting the three-component vector field data. By installing a number of these sensors on a rod and collecting data at several depth steps within the well, we are able to collect a 2D grid of three-component magnetic data along a single azimuth plane across the wellbore (Figure 4C). Additional rods installed either parallel to the primary concentric rod (Figure 4D), across other azimuthal directions (Figure 4E), or in some combination thereof (Figure 4F) will increase the spatial density of collected data within the wellbore.

**Figure 5:**
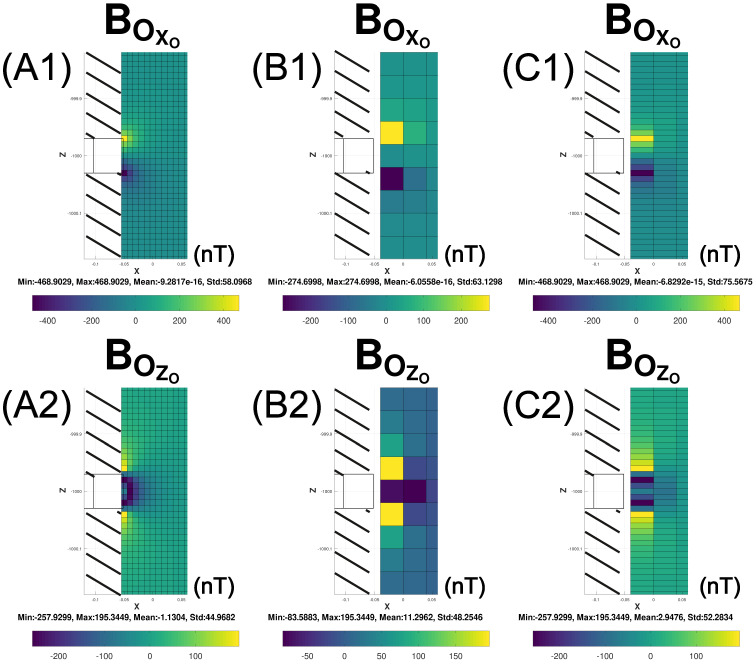
Three scenarios of ${B_{O}}_{X_{O}}$ and ${B_{O}}_{Z_{O}}$ GLQ modeling (8×8×8 nodes) for a formation washout in a magnetic layer of $M_{S}(M_{S_{X}}=0.0,M_{S_{Y}}=0.0,M_{S_{Z}}=-1.0$ A/m), extending from ${\rho_{S}}_{1}=0.06$ m to ${\rho_{S}}_{2}=0.12$ m, ${\phi_{S}}_{1}=150{}^{\circ}$ to ${\phi_{S}}_{2}=210{}^{\circ}$, and ${Z_{S}}_{1}=-1000.03$ m to ${Z_{S}}_{2}=-999.97$ m along the respective radial, azimuthal, and axial dimensions. Data are modeled and displayed on grids of (**A1**,**A2**) 0.01 m×0.01 m, (**B1**,**B2**) 0.04 m×0.04 m and (**C1**,**C2**) 0.04 m×0.01 m.

To ensure that a magnetic source is properly detected in the vicinity of a borehole wall, the sensors should not only be able to capture the magnitude of the anomaly signals above the noise level but also be able to capture the spatial variations of the anomaly signals within the borehole environment. According to Equations (4), (6) and (7), the magnetic effects of a differential source body at an observation point is a multi-variable function of the body’s volume ($dV_{S}$) and magnetization vector ($M_{S}$) plus the $R_{\mathrm{SO}}$ vector connecting the body to the observation point. The magnetic signature of an extended source body, on the other hand, is the sum of the magnetic effects of all differential source prisms that fill out the body (Equation (8)). Therefore, the magnetic anomaly for a borehole magnetic source is directly affected by the size and shape of the source, the relative distances between the observation points and the source, and intensity of magnetization.

The proper detection of a magnetic source also requires the sampling of the magnetic anomaly to be sufficiently dense, enabling the capture of the anomaly’s spatial variation details and suppressing the horizontal and vertical aliasing [16]. Vertical aliasing is mitigated by setting the maximum vertical sampling length dZ (Figure 4C) to half the length of the anticipated anomaly’s dominant vertical wavenumber (Nyquist wavenumber). The horizontal dimensions of the sensor relative to the diameter of the borehole, however, are often critical for mapping the magnetic anomaly. The diameter of petroleum exploration and production wells, for example, commonly range from ∼0.1 m to ∼0.5 m. Mining and engineering applications, on the other hand, can involve larger diameter wells up to ∼3.7 m [17].

Sensor size is not of a critical issue for the wider wells. However, for small wells, the size of the sensor is important as it determines how many observations can be acquired across the wellbore’s diameter. To help suppress horizontal aliasing, small-diameter magnetic sensors are available (Table A5) for effectively sampling across the diameter of the well. In this regard, Equation (6) facilitates determining the ranges of horizontal and vertical sampling rates for a sufficient sampling of the magnetic anomaly from the anticipated magnetic source. Magnetic source mapping also requires that the anomaly’s amplitudes fall within the detectable range of the magnetic sensors. Modern sensors routinely detect fields values as small as 0.1 nT (Table A5). Here again, Equation (6) can provide insight on the sensor’s capacity for mapping the magnetic anomaly of the anticipated source.

Finally, we examine how the size of the observation grid affects the imaging of the formation washout’s magnetic anomalies. Figure 5 illustrates the GLQ simulations (8×8×8 nodes) of the ${B_{O}}_{X_{O}}$ and ${B_{O}}_{Z_{O}}$ components for a formation washout in a magnetic layer of $M_{S}(M_{S_{X}}=0.0,\;M_{S_{Y}}=0.0,\;M_{S_{Z}}=-1.0$ A/m), extending from ${\rho_{S}}_{1}=0.06$ m to ${\rho_{S}}_{2}=0.12$ m, ${\phi_{S}}_{1}=150{}^{\circ}$ to ${\phi_{S}}_{2}=210{}^{\circ}$, and ${Z_{S}}_{1}=-1000.03$ m to ${Z_{S}}_{2}=-999.97$ m along the respective radial, azimuthal, and axial dimensions.

In the first scenario (A1 and A2), the data are modeled and displayed on a grid spacing of dX=0.01 m×dZ=0.01 m. Although the anomalies clearly infer the presence of a magnetic source such as a possible formation washout, this scenario may be impractical because the grid spacing is smaller than the horizontal dimension of the sensor (0.04 m).

**Figure 6:**
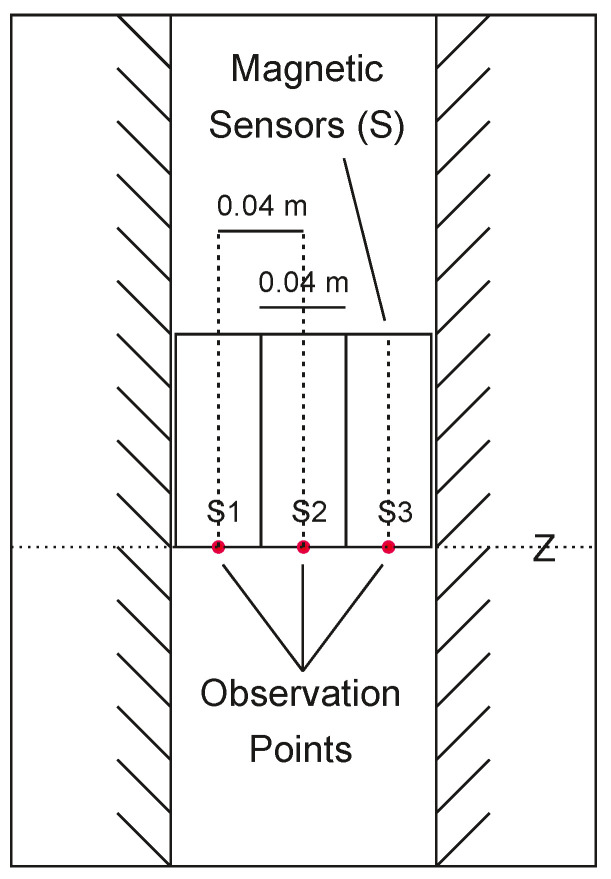
Layout of the second (**B1**, **B2**) and third (**C1**, **C2**) GLQ modeling simulations of Figure 5, where only three sensors of horizontal dimension equal to 0.04 m are installed on a rod within a well of diameter ≃0.12 m.

In the second scenario (B1 and B2), the magnetic effects are modeled and displayed at the more realistic grid spacing of dX=0.04 m×dZ=0.04 m. Accordingly, only three 0.04 m-diameter sensors are arrayed across the 0.12 m-diameter well (Figure 6). Here, the results show that increasing the grid spacings decreases the horizontal and vertical anomaly resolutions.

Finally, scenario three (C1 and C2) considers improving the vertical anomaly resolution by decreasing the grid spacing along the borehole’s axial dimension. This scenario, accordingly, models and displays data at a grid spacing of dX=0.04 m×dZ=0.01 m. In general, the magnetic anomaly amplitude variations for this and the other two scenarios are all in the ±100 nT range that modern magnetic sensors readily detect.

These simulations predict that magnetic data collected across the borehole environment with small readily available modern sensors can generally detect small magnetic sources within or near the well. Customizing the probe by removing the sensors’ covers and installing the electronics in an encapsulated sensor array or using smaller-size MEMS sensors [18] decreases the grid spacing to improve anomaly resolution. Finally, interpolating between data ($A_{0}$ and $C_{0}$) collected along off-diameter rods ($A_{1}A_{2}$ and $C_{1}C_{2}$) running parallel to a diameter ($B_{1}B_{2}$) will improve the resolution of the data display along the diameter of the wellbore at $B_{0}$ (Figure 4D).

## 4. Discussion

### 4.1. Comparing GLQ Magnetic Modeling Against Other Magnetic Modeling Methods

This section contrasts the eight methods for magnetic modeling in cylindrical coordinates listed in Table 1. To carry out the FEM modeling, the Magnetic Field No Current (MFNC) Module of the Comsol software 6.0 [19] was used. The other seven methods were coded in the MATLAB R2022a 64-bit (win64) programming language [20].

The SMP method provides closed-form solutions for the three magnetic vector field components to test against component estimates from the other methods. Furthermore, the LVC and SA methods only model the $B_{O_{Z_{O}}}$ component of the homogeneous, vertically magnetized finite cylinder. Thus, the $B_{O_{Z_{O}}}$ estimates from the eight methods were compared for the finite cylinder with vertical magnetization. In addition, computational instabilities/singularities were assessed as functions of the cylinder’s dimensions and relative distance to the observation point. When necessary, we have updated equations to have SI units.

**Figure A7:**
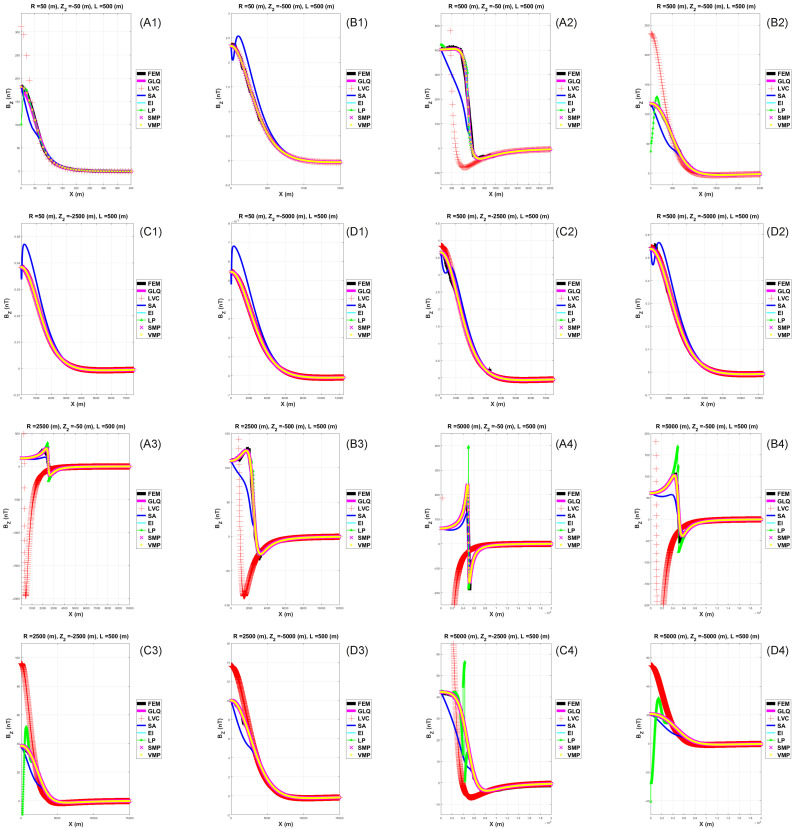
Modeled $B_{O_{Z_{O}}}$ component of vertical cylinders with a common length of L=500 m and varying radii and top depths of (**A1**,**B1**,**C1**,**D1**) R=50 m, (**A2**,**B2**,**C2**,**D2**) R=500 m, (**A3**,**B3**,**C3**,**D3**) R=2500 m and (**A4**,**B4**,**C4**,**D4**) R=5000 m, and (**A1**,**A2**,**A3**,**A4**) $Z_{2}=-50$ m, (**B1**,**B2**,**B3**,**B4**) $Z_{2}=-500$ m, (**C1**,**C2**,**C3**,**C4**) $Z_{2}=-2500$ m and (**D1**,**D2**,**D3**,**D4**) $Z_{2}=-5000$ m, respectively.

**Figure A8:**
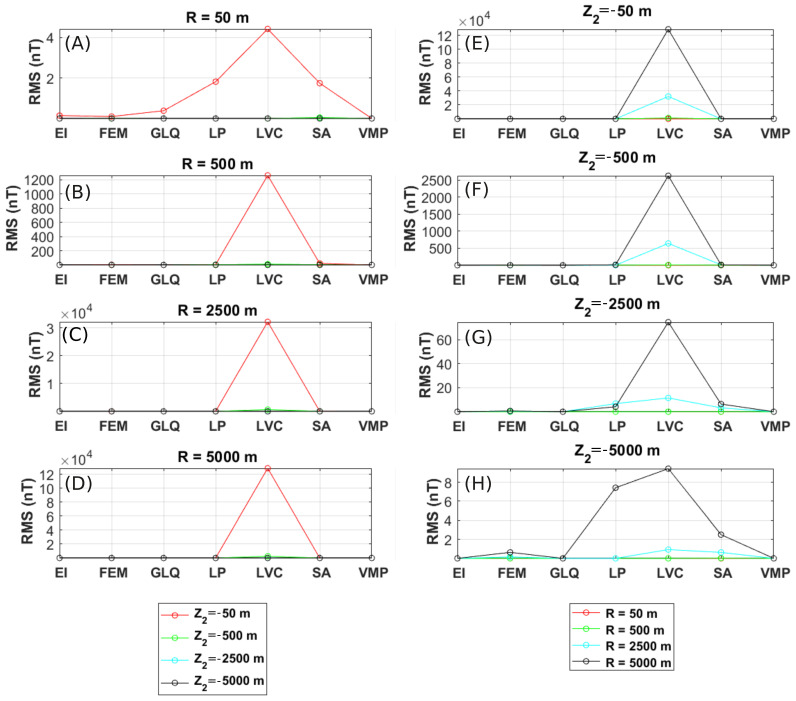
RMS differences between the SMP and the other 7 methods for the 16 modeled scenarios displayed in Figure A7. (**A**–**D**) correspond to a constant radius of R=50 m, R=500 m, R=2500 m and R=5000 m, whereas (**E**–**H**) correspond to a constant top depth of $Z_{2}=-50$ m, $Z_{2}=-500$ m, $Z_{2}=-2500$ m and $Z_{2}=-5000$ m below the surface (Z=0).

Figure A7 displays the $B_{O_{Z_{O}}}$ effects for the cylinder with magnetization attributes $M_{S}(M_{S_{X}}=0.0,\;M_{S_{Y}}=0.0,\;M_{S_{Z}}=1.0$ A/m); length L=500 m; radii R=50 m, 500 m, 2500 m, and 5000 m; and top-depths $Z_{2}=-50$ m, −500 m, −2500 m and −5000 m along the *X*-axis, with the station spacing of δX=10 m and extending from $X_{1}=0$ m to $X_{2}$ = 20,000 m at the elevation of Z = 0 m. The variations between curves are quantified by the Root Mean Square (RMS) differences between the SMP estimates and those of the other seven methods for the 16 modeling scenarios (Figure A8).

Figure A7 shows the eight modeled $B_{O_{Z_{O}}}$’s for the 16 scenarios. To better illustrate the details of the $B_{O_{Z_{O}}}$ estimates at the edges of the cylinders, the upper range of *X* ($X_{2}$) of each profile is selected differently. The figures suggest that the FEM, GLQ, EI, and VMP estimates are in very good agreement with those of the SMP method. However, the LP estimates deviate from the SMP estimates at near offsets when $1\leq \frac{R}{Z_{2}}\leq 2$, the LVC estimates deviate in near offsets from the SMP estimates when $\frac{R}{Z_{2}}>0.2$, and the SA estimates deviate from the SMP estimates at near offsets when $\frac{R}{Z_{2}}<100$.

In summary, the RMS results, as presented in Figure A8, showed that the LP, LVC, and SA estimates deviated the most from the SMP closed-form estimates. This assessment invoked the following methodology criteria.

1. For the Comsol software, the parameter selections included the ’Extremely Fine’ mesh size for maximum accuracy and the ’Tetrahedra’ mesh shape for maximum flexibility, whereas all other parameters were set at the software defaults. The volume of the modeling region was defined in the X, Y, and Z dimensions to range from −20,000 m to 20,000 m.

2. For the GLQ method, any desired number of nodes may be selected to fill in the source region. To ensure sufficient accuracy, the numbers of nodes used in the radial dimensions included 4 (R = 50 m), 22 (R = 500 m), 102 (R = 2500 m), and 202 (R = 5000 m), whereas in the azimuthal dimension, they were 360 (R = 50 m), 360 (R = 500 m), 360 (R = 2500 m) and 720 (R = 5000 m), and 4 nodes were used in the length dimension, giving rise to a total of 16 scenarios.

3. For the LP method, n = 5 terms in the Legendre polynomial series were used to ensure sufficient accuracy in computing $B_{O_{Z_{O}}}$.

Finally, Table 1 summarizes the computation times for the eight methods. Several factors affect the rigorous comparison of the computation time. First, all computations should be carried out on a common machine. Second, all methods should be coded and compiled in a common programming language. Third, all codes should adhere to the same code-optimization standard. To satisfy the first condition, both the Comsol and the MATLAB programming languages were installed and run on the x64-based PC-system-type Asus machine with processor specifications that include 13 Gen Intel(R) Core(TM) i9-13900K, 3000 MHz, 24 Core(s), 32 Logical Processor, and 64 GB of installed physical memory (RAM). However, the second condition was not satisfied because the Comsol software had been compiled and therefore run as an executable file, whereas the MATLAB codes were not compiled to a lower-level language. Also, the third condition was not satisfied because the Comsol software and the MATLAB-based codes were developed by different developer groups.

Nevertheless, Table 1 offers a first-order summary of the relative computation times to compute $B_{O_{Z_{O}}}$ for the cylinder in scenario 16, where R = 5000 m and $Z_{2}=-5000$ m from the eight methods. The analysis identified trade-offs between computational time and accuracy for the FEM, GLQ, and LP methods. The computational times for the FEM and GLQ methods, in particular, depended strongly on the respective mesh size and shape, and the number of nodes used in the radial, azimuthal, and length dimensions. Thus, to help optimize FEM or GLQ modeling, tests to determine the optimal mesh size and shape or number of GLQ nodes may be required.

For this study, the testing of the GLQ nodes showed that the number of nodes can be reduced from 202 to 12 in the radial dimension and from 720 to 45 in the azimuthal dimension, with negligible differences in the estimated magnetic effects and the advantage of reducing the computation time from about 70 s to 0.7025 s. Similar tests may be carried out to reach an optimal number of nodes for the other 15 scenarios. Finally, the most time-consuming estimates of $B_{O_{Z_{O}}}$ in Table 1 are from the LP method. Here, the computation time depends on how many terms (*n*) are used in the Legendre polynomial series.

The fourth column of Table 1 summarizes the ratio of the computing times for the methods to the compute the time for the GLQ method. These ratios range from a maximum of about 75.7234 for the LP method to a minimum of about 0.0016 for the LVC estimates. The LVC and SA methods clearly require much less time to compute $B_{O_{Z_{O}}}$. These methods are, however, prone to computational errors within the near offsets and do not offer a complete set of equations to model the potential, $B_{O_{\rho_{O}}}$, $B_{O_{\phi_{O}}}$, or the tensor field components. In conclusion, the GLQ method of this study offers not only the advantage of assessing the magnetic potential and vector field components but also, for the first time, the gradient tensor components of the differential prism in cylindrical coordinates.

### 4.2. Magnetic Gradieometry Advantages for Borehole Magnetic Applications

Magnetic solutions derived from the inversion of data collected at multiple altitudes are more comprehensive relative to those from single-altitude data [12]. Sampling via boreholes allows for collecting geomagnetic field data from the proximity of deeper magnetic sources [22]. A major challenge to conventional borehole magnetometry, where a single measurement is taken at every depth point, is the inability to determine from the measurements the azimuthal directions of the magnetic sources around the borehole.

This challenge may be overcome by sampling the field at several points along the wellbore’s diameter at multiple *Z*-depths and constructing a 2D image of the borehole’s magnetic field (as shown e.g., in Figure 4C). Comparing this image to models generated by GLQ integration (e.g., Figure A5 and Figure A6) provides insights on the spatial distribution of the magnetic sources around the borehole. Furthermore, magnetic gradiometry facilitates the suppression of the Earth’s diurnal and ambient field effects and enhances the anomaly details of the subsurface sources [22].

## 5. Conclusions and Recommendations

The complete magnetic effects (i.e., potential, vector and gradient components) of a cylindrical unit volume element or prism were developed to accommodate geological studies formatted in cylindrical coordinates. A conceptual design was presented for a probe to collect borehole magnetic gradiometry data with the ability to determine the azimuthal directions of magnetic sources around the borehole. This cylindrical modeling formulation may also serve other science and engineering applications such as modeling the effects of magnetic molecules [23] and electrical/electronic components [24,25,26], controlling and shaping magnetic fluids [27], and directional drilling in the oil and gas industry [28]. Finally, these results may be extended to modeling the gravity and thermal effects of arbitrarily oriented tubular bodies by Poisson’s theorem [12].

## Figures and Tables

**Table 1:** The third column from left gives the time required for the modeling methods to compute $B_{O_{Z_{O}}}$ for a cylinder with dimensions of L=500 m, R=5000 m and $Z_{2}=-5000$ m along the X-axis from $X_{1}=0$ to $X_{2}=20,000$ m. The fourth column from the left gives the time as ratio of the computing time for the GLQ method.

ID	Computational Method	Computational Time (s)	$\frac{t_{\mathrm{Method}}}{t_{\mathrm{GLQ}}}$	Reference
EI	Elliptical Integral	0.3408	0.4339	[5]
FEM	Finite Element Modeling	21.0000	29.8932	[21]
GLQ	Gauss–Legendre Quadrature	0.7025	1.0000	-
LP	Legendre Polynomial	53.1957	75.7234	[2]
LVC	Long Vertical Cylinder	0.0011	0.0016	[1]
SA	Solid Angle	0.0017	0.0024	[1]
SMP	Scalar Magnetic Potential	1.9417	2.7640	[7]
VMP	Vector Magnetic Potential	2.8659	4.0794	[6]

## Data Availability

The original contributions presented in the study are included in the article; further inquiries can be directed to the corresponding authors.

## Appendix B. Tables

**Table A1:** Formulae for $N_{1}$, $N_{2}$, $N_{3}$ and *D* in Equation (6).

Component	Formula
$N_{1}$	$\left({M_{S}}_{X}cos\phi_{O}+{M_{S}}_{Y}sin\phi_{O}\right)R_{SO}^{2}-3\left(\rho_{O}-\rho_{S}cos\left(\phi_{O}-\phi_{S}\right)\right)$ $\left({M_{S}}_{X}{R_{SO}}_{X}+{M_{S}}_{Y}{R_{SO}}_{Y}+{M_{S}}_{Z}{R_{SO}}_{Z}\right)$
$N_{2}$	$\left(-{M_{S}}_{X}sin\phi_{O}+{M_{S}}_{Y}cos\phi_{O}\right)R_{SO}^{2}$ $-3\rho_{S}sin\left(\phi_{O}-\phi_{S}\right)\left({M_{S}}_{X}{R_{SO}}_{X}+{M_{S}}_{Y}{R_{SO}}_{Y}+{M_{S}}_{Z}{R_{SO}}_{Z}\right)$
$N_{3}$	${M_{S}}_{Z}R_{SO}^{2}-3({M_{S}}_{X}{R_{SO}}_{X}+{M_{S}}_{Y}{R_{SO}}_{Y}$ $+{M_{S}}_{Z}{R_{SO}}_{Z})\left(Z_{O}-Z_{S}\right)$
*D*	$R_{SO}^{5}$

**Table A2:** Formulae for the magnetic tensor gradient components of the matrix in Equation (7).

Component	Formula
${{dB_{SO}}_{\rho_{O}}}_{\rho_{O}}$	$\frac{\partial ({dB_{SO}}_{\rho_{O}})}{\partial \rho_{O}}$
${{dB_{SO}}_{\rho_{O}}}_{\phi_{O}}$	$\frac{1}{\rho_{O}}\left(\frac{\partial ({dB_{SO}}_{\rho_{O}})}{\partial \phi_{O}}-{dB_{SO}}_{\phi_{O}}\right)$
${{dB_{SO}}_{\rho_{O}}}_{Z_{O}}$	$\frac{\partial ({dB_{SO}}_{\rho_{O}})}{\partial Z_{O}}$
${{dB_{SO}}_{\phi_{O}}}_{\rho_{O}}$	$\frac{\partial ({dB_{SO}}_{\phi_{O}})}{\partial \rho_{O}}$
${{dB_{SO}}_{\phi_{O}}}_{\phi_{O}}$	$\frac{1}{\rho_{O}}\left(\frac{\partial ({dB_{SO}}_{\phi_{O}})}{\partial \phi_{O}}+{dB_{SO}}_{\rho_{O}}\right)$
${{dB_{SO}}_{\phi_{O}}}_{Z_{O}}$	$\frac{\partial ({dB_{SO}}_{\phi_{O}})}{\partial Z_{O}}$
${{dB_{SO}}_{Z_{O}}}_{\rho_{O}}$	$\frac{\partial ({dB_{SO}}_{Z_{O}})}{\partial \rho_{O}}$
${{dB_{SO}}_{Z_{O}}}_{\phi_{O}}$	$\frac{1}{\rho_{O}}\frac{\partial ({dB_{SO}}_{Z_{O}})}{\partial \phi_{O}}$
${{dB_{SO}}_{Z_{O}}}_{Z_{O}}$	$\frac{\partial ({dB_{SO}}_{Z_{O}})}{\partial Z_{O}}$

**Table A3:** Formulae for the partial differentials in Table A2.

Component	Formula
$\frac{\partial ({dB_{SO}}_{\rho_{O}})}{\partial \rho_{O}}$	$-\frac{\mu_{0}\rho_{S}}{4\pi }\frac{{\left(N_{1}\right)}_{\rho_{O}}^{\prime }D-D_{\rho_{O}}^{\prime }N_{1}}{D^{2}}d\rho_{S}d\phi_{S}dZ_{S}$
$\frac{\partial ({dB_{SO}}_{\rho_{O}})}{\partial \phi_{O}}$	$-\frac{\mu_{0}\rho_{S}}{4\pi }\frac{{\left(N_{1}\right)}_{\phi_{O}}^{\prime }D-D_{\phi_{O}}^{\prime }N_{1}}{D^{2}}d\rho_{S}d\phi_{S}dZ_{S}$
$\frac{\partial ({dB_{SO}}_{\rho_{O}})}{\partial Z_{O}}$	$-\frac{\mu_{0}\rho_{S}}{4\pi }\frac{{\left(N_{1}\right)}_{Z_{O}}^{\prime }D-D_{Z_{O}}^{\prime }N_{1}}{D^{2}}d\rho_{S}d\phi_{S}dZ_{S}$
$\frac{\partial ({dB_{SO}}_{\phi_{O}})}{\partial \rho_{O}}$	$-\frac{\mu_{0}\rho_{S}}{4\pi }\frac{{\left(N_{2}\right)}_{\rho_{O}}^{\prime }D-D_{\rho_{O}}^{\prime }N_{2}}{D^{2}}d\rho_{S}d\phi_{S}dZ_{S}$
$\frac{\partial ({dB_{SO}}_{\phi_{O}})}{\partial \phi_{O}}$	$-\frac{\mu_{0}\rho_{S}}{4\pi }\frac{{\left(N_{2}\right)}_{\phi_{O}}^{\prime }D-D_{\phi_{O}}^{\prime }N_{2}}{D^{2}}d\rho_{S}d\phi_{S}dZ_{S}$
$\frac{\partial ({dB_{SO}}_{\phi_{O}})}{\partial Z_{O}}$	$-\frac{\mu_{0}\rho_{S}}{4\pi }\frac{{\left(N_{2}\right)}_{Z_{O}}^{\prime }D-D_{Z_{O}}^{\prime }N_{2}}{D^{2}}d\rho_{S}d\phi_{S}dZ_{S}$
$\frac{\partial ({dB_{SO}}_{Z_{O}})}{\partial \rho_{O}}$	$-\frac{\mu_{0}\rho_{S}}{4\pi }\frac{{\left(N_{3}\right)}_{\rho_{O}}^{\prime }D-D_{\rho_{O}}^{\prime }N_{3}}{D^{2}}d\rho_{S}d\phi_{S}dZ_{S}$
$\frac{\partial ({dB_{SO}}_{Z_{O}})}{\partial \phi_{O}}$	$-\frac{\mu_{0}\rho_{S}}{4\pi }\frac{{\left(N_{3}\right)}_{\phi_{O}}^{\prime }D-D_{\phi_{O}}^{\prime }N_{3}}{D^{2}}d\rho_{S}d\phi_{S}dZ_{S}$
$\frac{\partial ({dB_{SO}}_{Z_{O}})}{\partial Z_{O}}$	$-\frac{\mu_{0}\rho_{S}}{4\pi }\frac{{\left(N_{3}\right)}_{Z_{O}}^{\prime }D-D_{Z_{O}}^{\prime }N_{3}}{D^{2}}d\rho_{S}d\phi_{S}dZ_{S}$

**Table A4:** Formulae for ${\left(N_{m}\right)}_{n}^{\prime }$s and $D_{n}^{\prime }$s in Table A3 where m∈{1,2,3} and $n\in \{\rho_{O},\phi_{O},Z_{O}\}$.

Component	Formula
${\left(N_{1}\right)}_{\rho_{O}}^{\prime }$	$-\left(\rho_{O}-\rho_{S}cos\left(\phi_{O}-\phi_{S}\right)\right)\left({M_{S}}_{X}cos\phi_{O}+{M_{S}}_{Y}sin\phi_{O}\right)-3\left({M_{S}}_{X}{R_{SO}}_{X}+{M_{S}}_{Y}{R_{SO}}_{Y}+{M_{S}}_{Z}{R_{SO}}_{Z}\right)$
${\left(N_{1}\right)}_{\phi_{O}}^{\prime }$	$\left(R_{SO}^{2}-3\rho_{O}\left(\rho_{O}-\rho_{S}cos\left(\phi_{O}-\phi_{S}\right)\right)\right)\left(-{M_{S}}_{X}sin\phi_{O}+{M_{S}}_{Y}cos\phi_{O}\right)+2\rho_{O}\rho_{S}sin\left(\phi_{O}-\phi_{S}\right)\left({M_{S}}_{X}cos\phi_{O}+{M_{S}}_{Y}sin\phi_{O}\right)-3\rho_{S}sin\left(\phi_{O}-\phi_{S}\right)\left({M_{S}}_{X}{R_{SO}}_{X}+{M_{S}}_{Y}{R_{SO}}_{Y}+{M_{S}}_{Z}{R_{SO}}_{Z}\right)$
${\left(N_{1}\right)}_{Z_{O}}^{\prime }$	$2\left(Z_{O}-Z_{S}\right)\left({M_{S}}_{X}cos\phi_{O}+{M_{S}}_{Y}sin\phi_{O}\right)-3\left(\rho_{O}-\rho_{S}cos\left(\phi_{O}-\phi_{S}\right)\right){M_{S}}_{Z}$
${\left(N_{2}\right)}_{\rho_{O}}^{\prime }$	$2\left(\rho_{O}-\rho_{S}cos\left(\phi_{O}-\phi_{S}\right)\right)\left(-{M_{S}}_{X}sin\phi_{O}+{M_{S}}_{Y}cos\phi_{O}\right)-3\rho_{S}sin\left(\phi_{O}-\phi_{S}\right)\left({M_{S}}_{X}cos\phi_{O}+{M_{S}}_{Y}sin\phi_{O}\right)$
${\left(N_{2}\right)}_{\phi_{O}}^{\prime }$	$-R_{SO}^{2}\left({M_{S}}_{X}cos\phi_{O}+{M_{S}}_{Y}sin\phi_{O}\right)-\rho_{O}\rho_{S}sin\left(\phi_{O}-\phi_{S}\right)\left(-{M_{S}}_{X}sin\phi_{O}+{M_{S}}_{Y}cos\phi_{O}\right)-3\rho_{S}cos\left(\phi_{O}-\phi_{S}\right)\left({M_{S}}_{X}{R_{SO}}_{X}+{M_{S}}_{Y}{R_{SO}}_{Y}+{M_{S}}_{Z}{R_{SO}}_{Z}\right)$
${\left(N_{2}\right)}_{Z_{O}}^{\prime }$	$2\left(Z_{O}-Z_{S}\right)\left(-{M_{S}}_{X}sin\phi_{O}+{M_{S}}_{Y}cos\phi_{O}\right)-3\rho_{S}sin\left(\phi_{O}-\phi_{S}\right){M_{S}}_{Z}$
${\left(N_{3}\right)}_{\rho_{O}}^{\prime }$	$2\left(\rho_{O}-\rho_{S}cos\left(\phi_{O}-\phi_{S}\right)\right){M_{S}}_{Z}-3\left({M_{S}}_{X}cos\phi_{O}+{M_{S}}_{Y}sin\phi_{O}\right)\left(Z_{O}-Z_{S}\right)$
${\left(N_{3}\right)}_{\phi_{O}}^{\prime }$	$2\rho_{O}\rho_{S}sin\left(\phi_{O}-\phi_{S}\right){M_{S}}_{Z}-3\rho_{O}\left(Z_{O}-Z_{S}\right)\left(-{M_{S}}_{X}sin\phi_{O}+{M_{S}}_{Y}cos\phi_{O}\right)$
${\left(N_{3}\right)}_{Z_{O}}^{\prime }$	$-\left(Z_{O}-Z_{S}\right){M_{S}}_{Z}-3\left({M_{S}}_{X}{R_{SO}}_{X}+{M_{S}}_{Y}{R_{SO}}_{Y}+{M_{S}}_{Z}{R_{SO}}_{Z}\right)$
${\left(D\right)}_{\rho_{O}}^{\prime }$	$5\left(\rho_{O}-\rho_{S}cos\left(\phi_{O}-\phi_{S}\right)\right)R_{SO}^{3}$
${\left(D\right)}_{\phi_{O}}^{\prime }$	$5\rho_{O}\rho_{S}sin\left(\phi_{O}-\phi_{S}\right)R_{SO}^{3}$
${\left(D\right)}_{Z_{O}}^{\prime }$	$5\left(Z_{O}-Z_{S}\right)R_{SO}^{3}$

**Table A5:** Specifications of the magnetometers used to collect borehole magnetic data. The notations R, A, OT, D, L, M, P and F stand for Resolution, Accuracy, Operating Temperature, Diameter, Length, Mass of instrument, Proton and Fluxgate.

Company Name	Company Location	Product Name	R (nT)	A (nT)	OT (°C)	Dimension (m)	M (Kg)	Sensor Type
Aidu	China	ACZ-8	0.05	0.01	−10 to +50	0.0750 (D) × 0.1600 (L)	4	P
Chongqing Gold M&E Equipment	China	WCZ-3	0.05	0.5	−10 to +50	0.2060 × 0.0850 × 0.1550	1.5	P
Foerster	Germany	FEREX 4.035	-	-	−37 to +71	0.0394 (D) × 0.7500 (L)	3.3	F
GEM	Canada	GEM-GSM-19T	0.01	0.1	−40 to +50	0.2230 × 0.0690 × 0.2400	2.2	P
Geomative	China	GPM 10	0.01	0.1	−40 to +55	0.0700 (D) × 0.1400 (L)	1.0	P
Geomatrics	UK	G-857	0.1	0.5	0 to +40	0.1800 × 0.0900 × 0.2700	2.7	P
Satisgeo	Czech Republic	PMG-2	0.01	0.5	−10 to +60	0.0800 (D) × 0.2000 (L)	0.7	P
Sensys Magnetometer	Germany	FGM650	<0.2	-	−20 to +70	0.0350 (D) × 0.8500 (L)	0.72	F
SPECTOR Sci-tech LLC	Russia	TRITON	0.1	-	−20 to +70	0.1200 × 0.0600 × 0.0300	0.42	F
STL System	Switzerland	DM-050	0.002	-	−5 to +50	0.0480 (D) × 0.1500 (L)	0.3	F
Terra plus	Canada	GSM-19TGW4	0.01	0.1	−40 to +50	0.2230 × 0.0690 × 0.2400	2.2	P
Vallon	Germany	VSM4	<0.1	-	−38 to +71	0.0320 (D) × 0.7050 (L)	0.55	F

## Appendix C. Workflow for GLQ Modeling

We present a workflow for computing the magnetic effects of a source at a series of prescribed observation points in any natural coordinate system. In step one, the volume of the magnetic body is approximated using a series of infilling prisms. Smaller prisms capture more details about the source body’s shape. However, a larger number of prisms is needed here to infill the source body. Larger prisms may be used instead at the expense of decreasing the accuracy of source volume approximation. In steps two and three, for every prism, the number of nodes and associated coefficients are first selected, and next, the nodes are adjusted to the prism’s dimensions. In step four, the magnetic effects of the prism are computed at all prescribed observation points. Steps two to four are repeated for all source prisms. Finally, the computed effects of all prisms at every observation point are summed accordingly with the process repeated for all observation points to arrive at the magnetic field estimates of the source body across the entire observational domain.
